# CGF-Conditioned Medium Modulates Astrocytic Differentiation and Invasiveness in U87MG Glioblastoma Cells

**DOI:** 10.3390/biology14101461

**Published:** 2025-10-21

**Authors:** Laura Giannotti, Benedetta Di Chiara Stanca, Francesco Spedicato, Christian Demitri, Eleonora Stanca, Andrea Palermo, Franco Ferrante, Fabrizio Damiano, Maria Antonietta De Sangro, Luciano Abbruzzese, Luisa Siculella

**Affiliations:** 1 Department of Experimental Medicine, University of Salento, 73100 Lecce, Italy; christian.demitri@unisalento.it (C.D.); eleonora.stanca@unisalento.it (E.S.); andrea.palermo2004@libero.it (A.P.); fabrizio.damiano@unisalento.it (F.D.); luisa.siculella@unisalento.it (L.S.); 2 Institute of Polymers, Composites and Biomaterials, National Research Council (IPCB-CNR), 80125 Naples, Italy; 3 Department of Biological and Environmental Sciences and Technologies, University of Salento, 73100 Lecce, Italy; francesco.spedicato@unisalento.it; 4 Specialist in Oral Surgery, Private Practitioner, 73100 Lecce, Italy; franco_ferrante@yahoo.it; 5 Immunohaematology and Transfusion Medicine Unit, Vito Fazzi Hospital, 73100 Lecce, Italy; madesangro@gmail.com (M.A.D.S.); labbruzzese65@gmail.com (L.A.)

**Keywords:** astrocyte-like phenotype, concentrated growth factors (CGF), glioblastoma differentiation, tumor plasticity, U87MG

## Abstract

**Simple Summary:**

Glioblastoma is one of the most aggressive brain tumors, known for its ability to change shape and behavior, which makes it very difficult to treat. In our study, we tested whether substances naturally present in blood, called growth factors, could influence the behavior of glioblastoma cells grown in the laboratory. To do this, we used a special preparation, obtained from whole venous blood, rich in these growth factors, and exposed tumor cells to it for over one week. In the first days, the treated cells appeared more active, but later their ability to multiply and move decreased. Morphologically, the tumor cells began to show features more similar to normal brain support cells, called astrocytes, which are less harmful than tumor cells. We also observed that proteins typically linked to tumor aggressiveness decreased, while proteins typical of normal brain cells increased. These findings suggest that natural blood-derived preparations may encourage glioblastoma cells to adopt a less aggressive state. This knowledge may help future research explore new, more natural strategies to reduce tumor aggressiveness and possibly improve therapeutic approaches for this devastating disease.

**Abstract:**

Background: Glioblastoma (GBM) is a highly aggressive tumor characterized by elevated plasticity and poor differentiation. Platelet-derived preparations such as Concentrated Growth Factors (CGF) are rich in bioactive molecules, but their effects on tumor biology remain underexplored. Methods: U87MG glioblastoma cells were cultured with a conditioned medium obtained from CGF over 14 days (CGF-CM). We analyzed cell viability, morphology, DNA integrity, migration, proliferation, and expression of astrocytic markers. Results: CGF-CM treatment induced early enhancement of cell viability, followed by decreased proliferation and reduced migration at later time points. Morphological analyses revealed astrocyte-like features. The expression of glial fibrillary acidic protein (GFAP), an astrocytic marker, and its α/δ isoform ratio increased over time, while GBM -GBM-associated markers, such as AQP-4 and S100B, were downregulated. Conclusions: Our findings demonstrate that CGF-CM modulates the phenotypic plasticity of U87MG cells and promotes differentiation toward an astroglial-like profile. These results provide a basis for future studies on the modulation of GBM aggressiveness using bioactive autologous derivatives.

## 1. Introduction

In tissue regeneration, excellent results have been achieved using autologous platelet concentrates (APCs) derived from the patient’s peripheral blood. APCs are obtained by simple physical methods based on centrifugation and separation. The product is a plasma sample containing a high concentration of platelets as well as cytokines and growth factors involved in cell proliferation [1,2].

In recent years, several generations of APC have been produced; the latest one is Concentrated Growth Factors (CGF) [3]. In its solid form, it consists of a dense fibrin network, a natural scaffold rich in platelets and growth factors, with great regenerative potential. Autologous CGF is biocompatible, readily available, and safe [4].

In recent years, CGF has been extensively studied as an autologous blood derivative, capable of promoting tissue regeneration, new blood vessel formation, cell mobility, and differentiation [5,6]. One of the many fields of action of CGF in regenerative medicine is dental implantology, thanks to its propensity to induce osteogenic differentiation [7,8].

In the study by Zurita et al., it is highlighted that Platelet Rich Plasma (PRP), a first-generation APC, can be effectively used in combination with neurotrophic factors and other bioactive substances to promote the differentiation of bone marrow stromal cells (BMSCs) towards a neuronal phenotype. Specifically, the addition of Nerve Growth Factor (NGF), Brain-Derived Neurotrophic Factor (BDNF), and retinoic acid to PRP creates a biochemical environment rich in signals that favor the transformation of BMSCs into neuronal cells [9]. Given this evidence, platelet-derived products could represent promising tools not only in regenerative medicine but also in the modulation of neural phenotypes in pathological contexts. Despite extensive application of CGF in tissue regeneration, little is known about its effects on pathological cellular contexts such as malignancies. In particular, whether CGF can modulate the phenotype and plasticity of tumor cells remains largely unexplored [4]. Investigating these effects may provide new insights into GBM biology, the most common and aggressive brain tumor in adults, which continues to have a poor prognosis despite conventional treatments such as surgery, radiotherapy, and chemotherapy [10,11,12].

According to the 2021 WHO classification of central nervous system tumors, gliomas are now defined not only by histology but also by molecular and genetic features, with GBM representing the most aggressive entity among them. GBM accounts for about 50% of malignant gliomas, is most frequently diagnosed in patients over 65 years of age, and remains incurable despite current therapies [11,12].

Several authors have tested the differentiation potential of a human GBM cell line, U87MG, obtaining positive results regarding differentiation into astrocytes using stimuli such as N-(4-Hydroxyphenyl) retinamide [13] or cAMP activators [14].

NGF and BDNF are both secreted by platelets and, together with various growth factors found in platelets, are known to have regenerative, antiapoptotic, and neuroprotective effects on the nervous system [15].

Interestingly, another class of growth factors connected with this field of application is the Bone morphogenetic proteins (BMPs). BMPs generally act as tumor suppressors in brain tumors by promoting differentiation and blocking cellular proliferation. BMPs inhibit the tumorigenic potential of human brain tumor-initiating cells and GBM stem-like cells, highlighting their role in suppressing tumor growth [16]. Remarkably, BMP4 treatment decreases the percentage of CD133+ cells in GBM cancer stem cells (CSCs) and promotes their differentiation towards astrocytes, reducing the stemness of the CSCs population [17]. Moreover, BMP2 has been shown to increase the sensitivity of GBM cells resistant to temozolomide (TMZ)-induced apoptosis. Additionally, when BMP2 and Temozolomide (TMZ) are used together, this combination has been found to promote differentiation, decrease HIF1α activity, and subsequently lower O-6-methylguanine-DNA methyltransferase expression, which usually confers tumor resistance to chemotherapy [18].

Recent studies have highlighted the ability of autologous platelet-rich preparations to promote neurogenic differentiation, neural repair, and regeneration [19,20]. Borsani et al. reported that CGF regulates the expression of neuronal markers and supports differentiation in SH-SY5Y neuroblastoma cells [21]. In addition, Panek et al. demonstrated that local treatment with Platelet-Rich Fibrin patches suppresses glioma progression by inducing apoptosis in glioma cells and modulating the tumor microenvironment in a murine model [22].

Despite these promising observations, no study has yet clarified how the secretome of CGF may influence the phenotype of GBM cells in vitro. Building on this background, CGF, thanks to its bioactive molecules and growth factors, may not only influence tumor cell proliferation and migration but also modulate phenotypic plasticity and differentiation, thereby contributing to the understanding of glioma biology. Consequently, this work aims to explore the effects of CGF-conditioned medium (CGF-CM) on U87MG glioblastoma cells, focusing on morphological changes, expression of differentiation markers, and cellular behaviors related to proliferation and migration. The study investigates the hypothesis that CGF-CM may induce astrocyte-like features in GBM cells, providing a model to study differentiation-based modulation of tumor aggressiveness.

## 2. Materials and Methods

### 2.1. Preparation of CGF and Conditional Medium

Blood samples (8 mL each) were collected via venipuncture from five healthy, non-smoking donors (3 males and 2 females, aged 25–45 years) who signed the informed consent. The inclusion criteria comprised the absence of acute or chronic diseases and no ongoing pharmacological treatments, while the exclusion criteria included pregnancy, infectious diseases, smoking, or the use of anti-inflammatory or anticoagulant drugs within two weeks prior to blood collection. For each set of experiments, CGF was prepared from the same blood sample of a single donor to ensure consistency, and samples from different donors were pooled to minimize donor-to-donor variability. Blood tubes were processed using a Medifuge MF200 device (Silfradent S.r.l., Forlì, Italy) according to the manufacturer’s instructions. CGF was added directly to 6-well plates for 14 days with DMEM low glucose (L0064-500, Voden medical instruments, Casorezzo (Mi), Italy), 1% (*v*/*v*) penicillin-streptomycin (P4333, Sigma-aldrich, Milan, Italy), and 2 mM L-glutamine (X0550-100, Voden medical instruments). Plates containing CGF were incubated in a humidified incubator containing 95% air and 5% CO_2_ at 37 °C. After 5, 9, and 14 days, the 100% medium was removed and placed in a 15 mL tube and centrifuged at 14,000 rpm for 1 min, obtaining the conditioned media, and fresh growth medium was added to the plate with the CGF. The content of specific growth factors, cytokines, and chemokines in the CGF-conditioned medium, including VEGF, PDGF, TGF-β, and BMP, has been previously characterized in our earlier study [6].

### 2.2. Cell Culture and Treatment

U87MG cells (HTB-14, ATCC, Rockville, MD, USA) were cultured in high-glucose DMEM (L0101-500, Voden medical instruments) with 10% fetal bovine serum (FBS, S181H-500, Voden medical instruments), 1% penicillin-streptomycin, and 2 mM L-glutamine. Cells were incubated at 37 °C in a humidified incubator with 95% air and 5% CO_2_. For all experiments, cells between passages 5 and 15 were used. At approximately 80% confluence, the cells were seeded in new dishes and treated with DMEM low glucose, 10% (*v*/*v*) FBS, 1% (*v*/*v*) penicillin-streptomycin and 2 mM L-glutamine using different percentages of the CGF conditioned medium (10%, 30%, and 50%) for the time treatment (1, 4, 7 days). A specific control was used in all experiments for each treatment condition and time point. The experimental parameters employed were as follows: cells in low glucose DMEM medium with 10% FBS only, which represents the control (CTR) cultured for 1, 4, and 7 days, 10% CGF-CM (cells treated with 10% conditioned medium for 1, 4, and 7 days), 30% CGF-CM (cells treated with 30% conditioned medium for 1, 4, and 7 days), 50% CGF-CM (cells treated with 50% conditioned medium for 1, 4, and 7 days). After the vitality test, all experiments are carried out considering only the 30% concentration of CGF-CM for a duration of 1, 4, and 7 days.

### 2.3. Cell Viability Assay

The MTT (3-(4,5-dimethylthiazol-2-yl)-2,5-diphenyltetrazolium bromide) assay was conducted to evaluate the viability of U87MG cells. 1500 cells were seeded in single 96-well plate wells for CTR, 10% GCF-CM, 30% GCF-CM, and 50% GCF-CM for 1, 4, and 7 days, respectively. Subsequently, 20 μL of MTT solution (10 mg/mL) was added to each well and incubated at 37 °C for 4 h. Following incubation, absorbance was measured at 570 nm using a Multiskan™ FC Microplate Photometer (Thermo Fisher Scientific, Rodano, Italy).

### 2.4. Scratch Wound Healing Assay

The cells were seeded at a density of 2.5 × 10^4^ cells/well on a 24-well plate to ensure confluence and the formation of a continuous monolayer. The cells were treated with CGF-CM for 1, 4, and 7 days. At the end of each time point, a straight scratch was made in the cellular monolayer using a sterile pipette tip to create a “wound.” Subsequently, cellular debris was gently removed with a phosphate-buffered saline (PBS) wash. Images were then acquired at time zero (t = 0 h) and after 24 h (t = 24 h). Data analysis was performed by measuring the residual wound area at each time interval, and the percentage of wound closure was calculated using image analysis ImageJ software (version 1.54f).

### 2.5. Real-Time PCR

U87MG cells were plated in 60 mm plates at a density of 9 × 10^4^ cells per well; the conditions analyzed were cells untreated (CTR) and treated with 30% CGF-CM for 1, 4, and 7 days. RNA was extracted from each condition using TrizolTM Reagent (ThermoFisher Scientific, Waltham, MA, USA), following the manufacturer’s protocol. Reverse transcription was performed using 1 μg of total RNA, random primers, and MultiScribe^®^ Reverse Transcriptase (CA94404, Applied Biosystems, Monza, Italy) in a 20 μL reaction according to the manufacturer’s instructions. Quantitative analysis of gene expression was carried out on a CFX Connect Real-Time system (Bio-Rad, Hercules, CA, USA) using SYBR Green technology (FluoCycle, Euroclone, Milan, Italy). Gapdh was used as an internal control for normalization. The specificity of PCR products was confirmed by melt curve analysis, and reactions were performed in triplicate using three independent sets of RNA. Primer sequences are listed in Table 1.

### 2.6. Cell Counting and Growth Curve Protocol

The cells were seeded at a density of 5 × 10^4^ cells/well in a 12-well plate under CTR and CGF-CM conditions for 1, 4, and 7 days. At the end of the treatment period, the cells were incubated with 0.25% trypsin for 5 min at 37 °C; the action of trypsin was then blocked with a low-glucose DMEM medium containing 10% FBS. Finally, 20 μL were taken for cell counting using Bürker counting chambers (BR718920-1EA, Sigma-aldrich). Cell counting was repeated for each time point (t = 0, t = 1, t = 4, t = 7) and each condition. The data were plotted on a graph, with time on the *x*-axis and the number of cells on the *y*-axis.

### 2.7. Comet Assay

After treatment, cells were harvested by trypsinization, washed in PBS, counted, and resuspended in low-melting point agarose (Invitrogen, Carlsbad, CA, USA) to obtain a concentration of 0.5 × 10^6^ cells/mL. This solution was used to drop a droplet onto a microscope slide covered with a layer of normal-melting-point agarose. Subsequently, the point where the droplet was placed was covered with a coverslip and allowed to solidify for 5 min at 4 °C. Subsequently, the coverslip was removed, and the slides were immersed in lysis solution (2.5 M NaCl, 10 mM Tris HCl pH 8, 100 mM EDTA, and 1% Triton X-100) overnight at 4 °C. The day after, the slides were incubated in an alkaline buffer (300 mM NaOH and 1 mM EDTA) for 30 min at 4 °C to allow DNA unwinding. After that, electrophoresis was performed at 20 V for 30 min at 4 °C. Then, the slides were gently washed with PBS and stained with Syber Green^®^ (Life Technologies, Foster City, CA, USA) 1:2000 in PBS for 5 min in the dark. DNA damage was evaluated under a fluorescent microscope (EVOSTM FLoid, Invitrogen, Waltham, MA, USA), and DNA fragmentation was determined by measuring % Tail DNA using ImageJ software (version 1.54f). At least 50 cells were counted in each of the three replicates.

### 2.8. Western Blot

U87MG cells were plated at a density of 1 × 10^6^ cells per 100 mm dish for each experimental condition. To obtain total cellular protein extracts for Western blot analysis, cells were harvested in the following buffer: 20 mM Tris-HCl (pH 8.0), 420 mM NaCl, 2 mM EDTA, 2 mM Na_3_VO_4_, and 1% (*v*/*v*) Nonidet P-40, supplemented with a protease inhibitor cocktail. Cell lysates underwent freeze/thaw cycles and were then centrifuged at 10,000 rpm for 10 min at room temperature. The supernatant was collected and stored at −80 °C until further use. Total protein concentrations were determined using the Bio-Rad protein assay kit, with lyophilized bovine serum albumin (BSA) as the standard. Equal amounts of protein were denatured at 96 °C for 5 min and separated on 10% (*w*/*v*) SDS-PAGE gels. The separated proteins were then transferred to a nitrocellulose membrane (Pall, East Hills, NY, USA) via electrophoresis. Equal protein loading was confirmed by Ponceau S staining. The membrane was blocked with 2.5% (*w*/*v*) non-fat dry milk in buffered saline for 1 h at room temperature. The blots were then incubated overnight at 4 °C with specific primary antibodies at a 1:1000 dilution. The primary antibodies used were anti-GFAP (sc-166481, Santa Cruz Biotechnology, Dallas, TX, USA) and anti-β-actin (sc-47778, Santa Cruz Biotechnology, Dallas, TX, USA), anti-cyclinD1 (sc-450, Santa Cruz Biotechnology, Dallas, TX, USA), anti-p-Rb (sc-377528, Santa Cruz Biotechnology, Dallas, TX, USA) and anti-Caspase-3 (sc-7148, Santa Cruz Biotechnology, Dallas, TX, USA).

Immune complexes were detected using appropriate peroxidase-conjugated secondary antibodies (anti-mouse A90-116P, Bethyl Laboratories, Montgomery, TX, USA) for 1 h at room temperature, and immunoreactive bands were visualized using an enhanced chemiluminescence detection kit (#1705061, Bio-Rad, Hercules, CA, USA). Densitometric analysis of the blots was performed using the ChemiDoc MP imaging system (Bio-Rad, Hercules, CA, USA). Primary antibodies were used at a dilution of 1:1000, and HRP-conjugated secondary antibodies at a dilution of 1:2000, according to the manufacturer’s instructions.

### 2.9. Immunohistochemistry and Hematoxylin-Eosin Staining

Cells were plated at a density of 20 × 10^4^ cells per well in a 12-well plate for each experimental condition. U87MG cells were fixed in 4% paraformaldehyde in PBS, pH 7.4, at room temperature for 15 min after being stained using either an immunohistochemistry procedure or Hematoxylin-Eosin staining. For the immunohistochemistry protocols, the cells were incubated with a mouse primary monoclonal antibody (mAb) anti-GFAP (sc-166481, Santa Cruz Biotechnology, Dallas, TX, USA) overnight at 4 °C. Then, they were properly incubated with peroxidase-conjugated secondary antibodies (anti-mouse A90-116P, Bethyl Laboratories, Montgomery, TX, USA) for 1 h at room temperature. To detect the formation of the antigen-Ab complex, the cells were incubated with 3,3-diaminobenzidine (D3939, Sigma-Aldrich). Instead, for the protocol Hematoxylin-Eosin staining, we used the solution of 0.1% in PBS at room temperature for 10 min. Primary antibodies were used at a dilution of 1:100, and HRP-conjugated secondary antibodies at a dilution of 1:500, according to the manufacturer’s instructions.

### 2.10. Dapi Assay

Cells were plated at a density of 10 × 10^4^ cells per well of a 24-well plate for each experimental condition. The cells were fixed in 4% paraformaldehyde in PBS, pH 7.4, at room temperature for 15 min and incubated in a 0.1% DAPI solution in PBS at room temperature for 5 min.

### 2.11. Statistical Analysis

Data were analyzed by one-way analysis of variance (ANOVA) followed by Tukey’s multiple comparisons test or the Bonferroni/Dunn post hoc test. Scratch wound healing assay data were analyzed by two-way analysis of variance (ANOVA) followed by Tukey’s multiple comparisons test. All experiments were repeated a minimum of three times, and results were presented as mean ± standard deviation (SD). The analysis was performed using GraphPad Prism 9.5 (GraphPad Software, Boston, MA, USA), and *p* < 0.05 was considered statistically significant compared to the relative control cells at the same time point.

## 3. Results

### 3.1. CGF-CM Modulates U87MG Cell Viability in a Concentration- and Time-Dependent Manner

**Figure 1:**
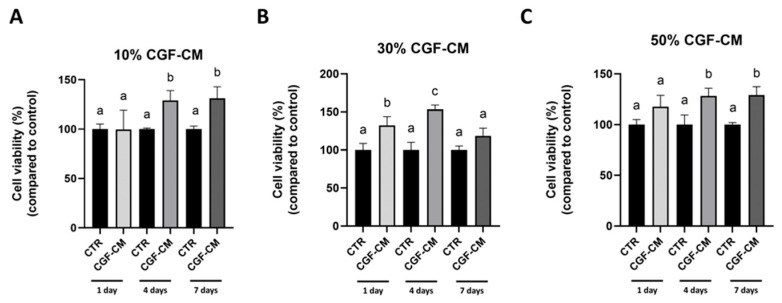
Effects of CGF-CM treatment on U87MG cell viability. U87MG cells were treated with CGF-CM at 10% (**A**), 30% (**B**), 50% (**C**), or untreated cells (CTR) for 1, 4, and 7 days. Viability was determined by MTT assay. Results are expressed as mean ± SD. Experiments were repeated independently in triplicate (*n* = 3), and samples with different letters were significantly different (*p* < 0.05) compared to the same time point control cells, whereas samples sharing the same letter were not significantly different.

To evaluate the cytotoxicity of the conditioned medium of CGF (CGF-CM) on the U87MG cell line, a cell viability analysis was performed at different time points (1, 4, 7 days) and at different concentrations of CGF-CM (10%, 30%, and 50%) using an MTT assay (Figure 1A–C). The treatment with CGF-CM induced multiple and diverse effects that varied depending on the duration of cell culture and the concentrations applied. Specifically, treatment with 10% and 50% CGF-CM led to a progressive increase in the viability of U87MG cells, which became evident starting from the 4th day of culture (Figure 1A,C). In contrast, Figure 1B shows that treatment with 30% CGF-CM produced a significant increase in cell viability already from the 1st day of incubation, indicating a more immediate response to this concentration. Notably, the most pronounced increase was observed at 4 days, highlighting a peak in the metabolic response to CGF-CM at this time point; however, after 7 days of culture, a decline in cell viability was noted.

Therefore, we observed that CGF-CM transiently enhances the metabolic activity of GBM cells in a concentration- and time-dependent manner, with a peak at 30% CGF-CM after 4 days.

To strengthen the reproducibility of our findings, we also carried out the same assay on the U251 GBM cells, which produced comparable results to those observed in U87MG cells (Supplementary Figure S1).

### 3.2. CGF-CM Does Not Cause DNA Damage

To ensure that U87MG cells remained in a healthy state following treatment with CGF-CM, we conducted targeted analyses to evaluate the absence of DNA damage. We aimed to comprehensively determine the safety profile of CGF-CM on this cell line by focusing on markers of DNA stability and damage.

**Figure 2:**
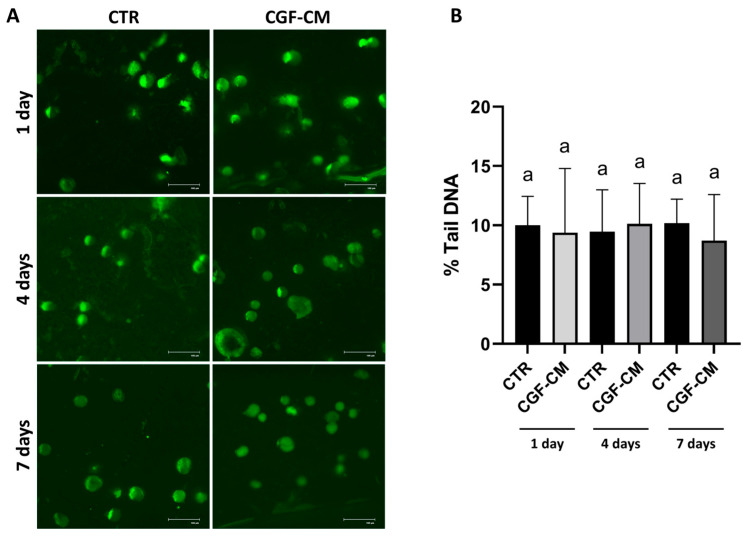
Effects of CGF-CM on DNA. (**A**) Comet assay images of cells treated with CGF-CM at different times (1, 4, and 7 days) and under control conditions (CTR) (scale bar: 100 μm); (**B**) Percentage of DNA in the tail analysis was assessed with ImageJ software (version 1.54f). The experiments were repeated three times independently (*n* = 3) and samples with different letters were significantly different (*p* < 0.05) compared with respect to respective control cells, whereas samples sharing the same letter were not significantly different.

Therefore, the comet assay was used to assess whether 30% CGF-CM treatment caused DNA damage at different time points. This assay allows the evaluation of single- and double-stranded DNA breaks. Figure 2A shows images taken under different conditions: no significant increase in tail length or percentage of DNA in the tail in cells treated with CGF-CM at different days, compared to each respective time point control (CTR), was observed, indicating that there was no increment in DNA damage. Statistical analysis of tail DNA showed no significant increase in the percentage of tail DNA (Figure 2A) compared to CTR. The percentage of tail DNA in the different conditions did not vary significantly and remained below 10% (Figure 2B).

**Figure 3:**
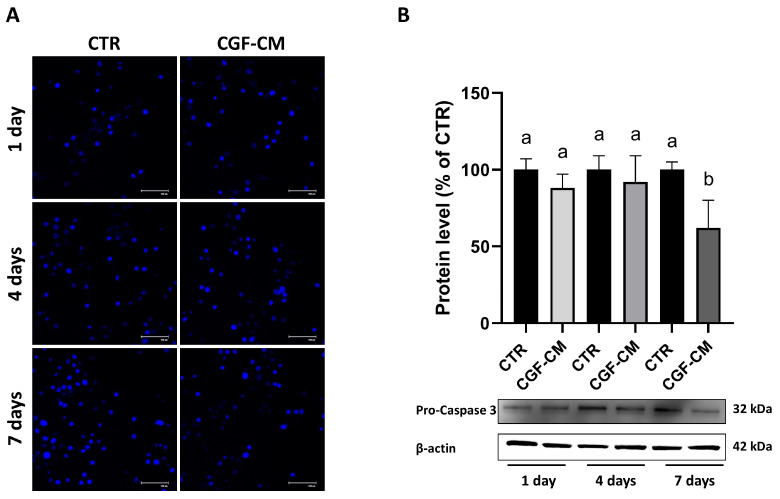
Absence of apoptosis in response to CGF-CM treatment. (**A**) U87MG cells stained with DAPI under different treatment conditions. Scale bar: 50 μm (**B**) Western blot analysis of Caspase-3 protein expression after treatment, using β-actin as a loading control. Experiments were repeated in triplicate independently (*n* = 3) and samples with different letters were significantly different (*p* < 0.05) compared to the respective control cells, whereas samples sharing the same letter were not significantly different.

For further confirmation, DAPI staining was performed, and the fluorescence microscope images (Figure 3A) showed intact nuclei in all experimental conditions analyzed. In the treated and control samples, the nuclei appeared round or oval with well-defined contours and uniform fluorescence distribution, indicating a regular distribution of nuclear DNA. There were no signs of apoptosis in the nuclei observed and no phenomena of DNA condensation, nuclear fragmentation, or the presence of apoptotic bodies, which would indicate ongoing apoptotic processes.

Finally, Western blot analysis (Figure 3B) showed that the active cleaved form of Caspase-3 was not present in any of the samples; only a reduction in pro-Caspase-3 levels was observed at 7 days, but without the presence of its active form.

These findings indicate that CGF-CM did not trigger apoptotic pathways or DNA damage in U87MG cells under the conditions tested, supporting a non-cytotoxic modulation of cell behavior.

### 3.3. Effects on U87MG Morphometric Parameters After Treatment with CGF-CM

**Figure 4:**
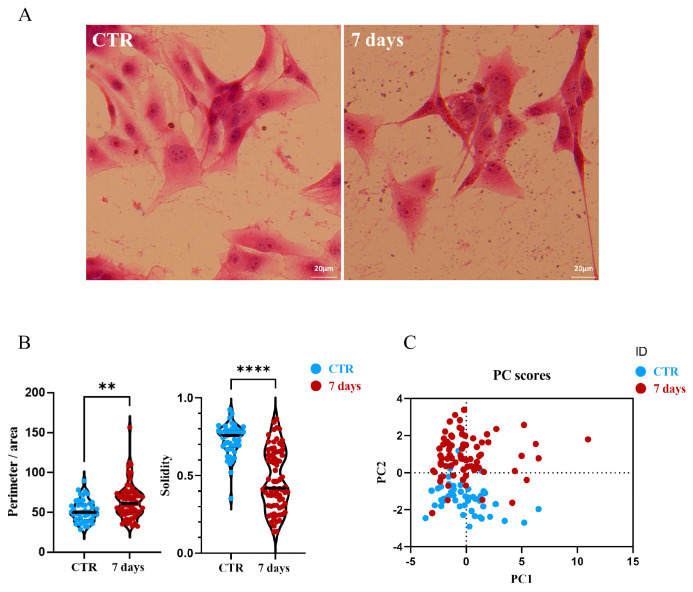
Effects of CGF-CM on morphometric parameters. (**A**) Micrographs of U87MG cells under CTR and 30% of CGF-CM conditions for 7 days. Scale bar 20 μm. (**B**) Analysis of the effects of CGF-CM on the distance between cells, analyzed using ImageJ software (version 1.54f). Morphometric analysis of solidity and the ratio between perimeter and area (black bars indicate the population mode). Experiments were repeated three times independently (*n* = 3). ** *p* < 0.05 and **** *p* < 0.001 compared to control. The data are representative of three independent experiments, expressed as mean ± SD. (**C**) PCA of morphometric analysis data of U87 30%-7d treated and untreated cell groups. For each experimental group, 60 < *n* < 80 cells were analyzed.

In the U87MG cells cultured for 7 days in the CTR and 30% CGF-CM conditions, we observed a morphological change after treatment. These morphological changes are reminiscent of astrocyte-like morphology and prompted further investigation into potential differentiation processes. So, we used hematoxylin and eosin staining to better highlight these changes. As reported in Figure 4A, the cells treated with CGF-CM showed a phenotypic change compared to the control condition. In fact, after treatment, the cells appeared to have a more regular and star-like shape with branched radial processes, suggesting an astrocyte-like structure. These data are supported by the measurement of morphometric parameters, such as solidity and ratio between perimeter and area (Figure 4B), performed using ImageJ software (version 1.54f). This allowed the evaluation of a variation in the morphology complexity and the size of the process, probably indicating a reduced invasive capacity of the GBM cells. Moreover, principal component analysis (PCA) loading plots, using morphological variables, confirmed the clustered separation between CTR and 30% CGF-CM 7-day treatment (Figure 4C).

### 3.4. Effects of CGF-CM on U87MG Invasiveness and Proliferation

**Figure 5:**
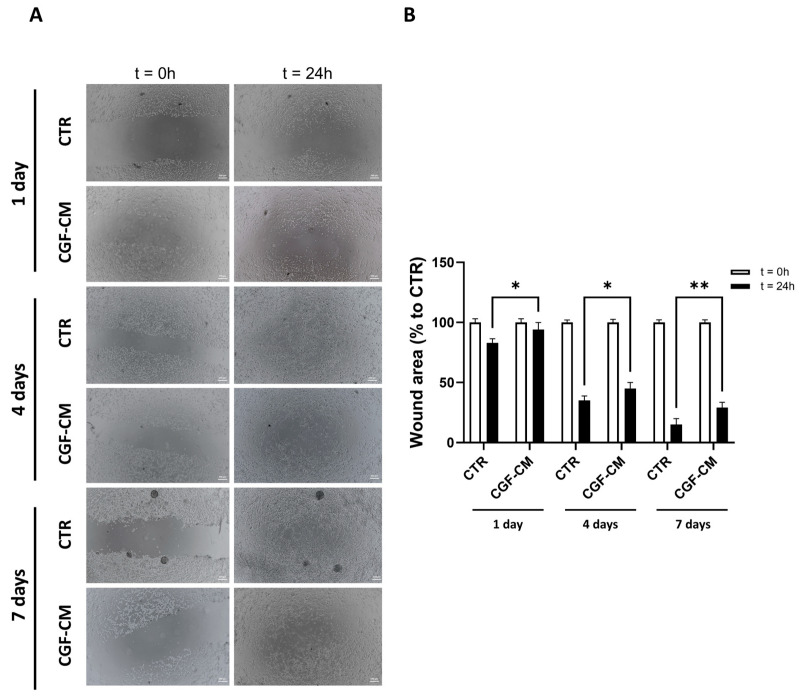
Cell invasion test and analysis. (**A**) Representative images of the wounds (scale bar: 200 μm) observed at different time points (t = 0 h and t = 24 h) under CTR and CGF-CM conditions at 1, 4, and 7 days. (**B**) Analysis of wound area in samples treated with CGF-CM and CTR. Results are expressed as mean ± SD; experiments were repeated three times independently (*n* = 3). * *p* < 0.05, and ** *p* < 0.01 compared to their relative control.

The hypothesis that morphological change might be related to changes in migration and proliferation led us to investigate these aspects further. Therefore, U87MG control cells and cells treated with 30% CGF-CM for 1, 4, and 7 days were analyzed for their migratory capacity through the wound healing assay and for cell cycle regulation through RT-PCR and growth curve analysis. Figure 5A shows images of the wounds observed at different time points (t = 0 h and t = 24 h). The closure of the wound indicates cell migration. In our results, the wounds in the controls healed faster than in the treated samples over the same period. The wound area data at 24 h (Figure 5B) demonstrated a less pronounced reduction in wound size in the samples treated with CGF-CM compared to the control at each analyzed time point—specifically at 1, 4, and 7 days. This indicates that CGF-CM reduced the migratory behavior of U87MG cells, as reflected by delayed wound closure. Specifically, the wound closure was consistently reduced across all time points (11% at 1 day, 10% at 4 days, and 15% at 7 days) compared to the controls.

**Figure 6:**
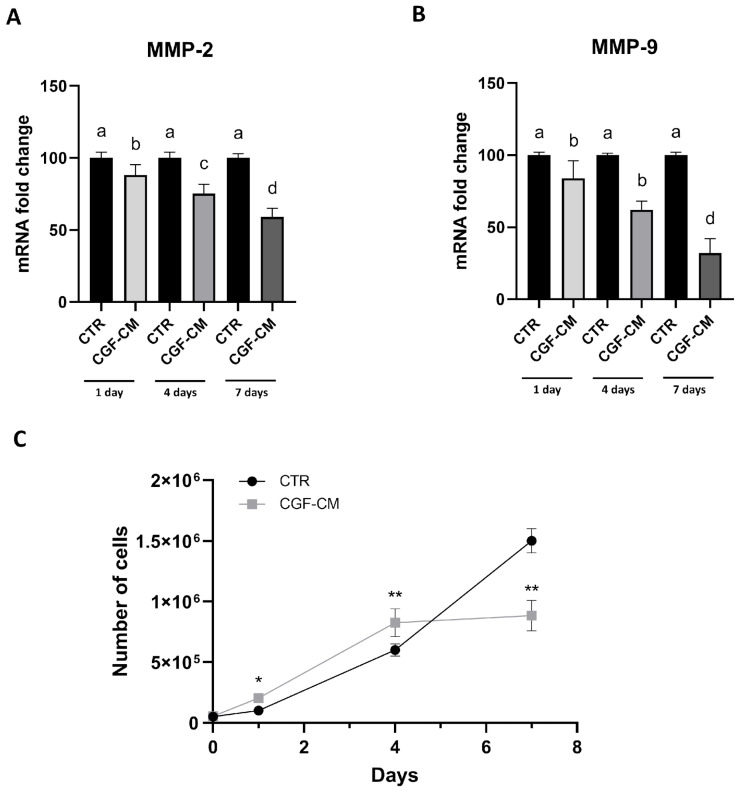
U87MG invasiveness after treatment with CGF-CM. (**A**) mRNA abundance of MMP-2 and (**B**) MMP-9 compared to the CTR. (**C**) Growth curve at 1, 4, and 7 days for CTR and CGF-CM; * *p* < 0.05, and ** *p* < 0.01 compared to the control. GAPDH was used for RT-PCR data normalization. Results are expressed as mean ± SD; experiments were repeated three times independently (*n* = 3), and samples with different letters are significantly different (*p* < 0.05) compared to their own control cells, whereas samples sharing the same letter are not significantly different.

To complement the wound healing assay, the mRNA levels of matrix metalloproteinases 2 and 9 (MMP-2, MMP-9), genes associated with cell invasiveness, were analyzed. As shown in Figure 6A,B, the expression levels of these genes were significantly decreased at all time points compared to the respective control (CTR).

Therefore, we also performed a growth curve analysis to evaluate cell proliferation in controls and treated samples at 1, 4, and 7 days. As shown in Figure 6C, the growth curves of cells treated with CGF-CM exhibited significant differences compared to the controls on the first and fourth days of treatment, indicating an initial acceleration of cell growth. At the 7-day time point, however, the trend reversed, with the treated samples showing a 59% reduction in cell number compared to the control.

**Figure 7:**
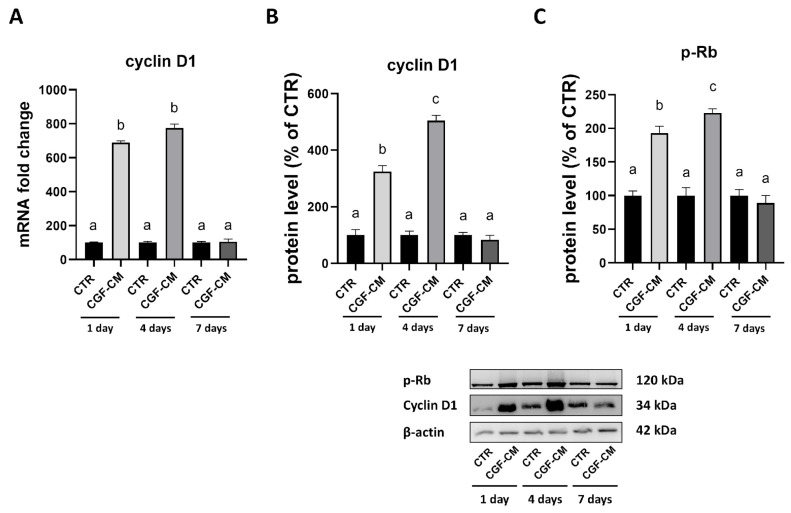
U87MG proliferation after treatment with CGF-CM. (**A**) mRNA levels of cyclin D1. GAPDH was used for RT-PCR data normalization. (**B**) Western blot analysis of cyclin D1 and (**C**) Rb protein expression after treatment, using β-actin as a loading control. Results are expressed as mean ± SD; experiments were repeated three times independently (*n* = 3), and samples with different letters are significantly different (*p* < 0.05) compared to the control cells, whereas samples sharing the same letter are not significantly different.

Based on the observed growth curve, where treatment with CGF-CM slowed the growth of U87MG cells compared to the control, the expression of cyclin D1 (Figure 7A), a key regulator of the cell cycle, was also analyzed. As reported in Figure 7A, the reduction in cyclin D1 gene expression over time in CGF-CM conditions is consistent with the previously described findings. Specifically, cyclin D1 gene expression increased compared to controls up to 4 days of treatment and then dramatically decreased at 7 days of treatment compared to controls. A similar trend was observed at the protein level (Figure 7B), with cyclin D1 peaking at day 4 and returning to baseline levels by day 7. This was further supported by analysis of phosphorylated Rb (p-Rb), another marker of cell cycle progression (Figure 7C). Levels of p-Rb followed a similar pattern, increasing significantly on day 4 and returning to control levels by day 7. These results suggest that the early upregulation of cyclin D1 and p-Rb upon CGF-CM treatment may drive an initial phase of cell cycle activation, followed by a subsequent decline in these markers, potentially contributing to the growth arrest observed at a successive time point.

### 3.5. CGF-CM Promotes Astrocyte-Like Differentiation Features in U87MG Cells

**Figure 8:**
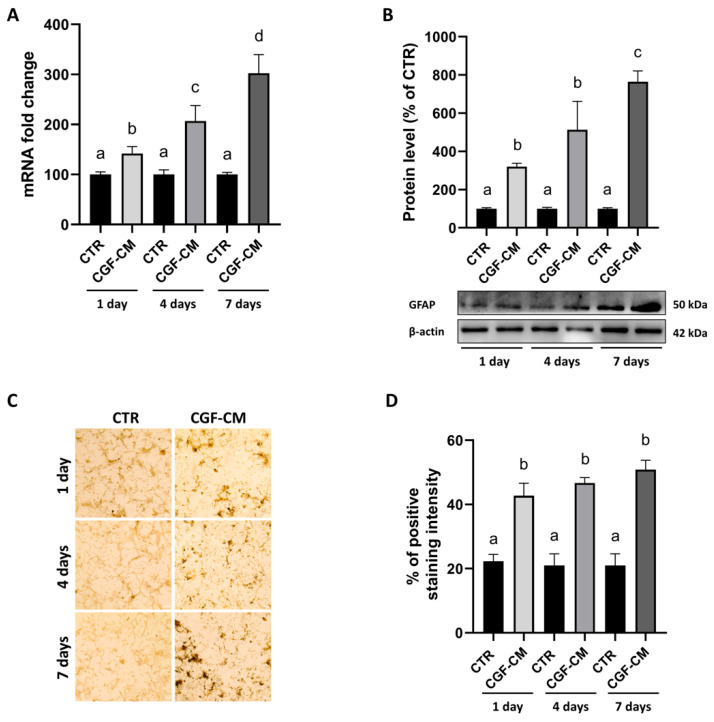
Increase in GFAP expression levels after treatment with CGF-CM. GFAP expression was analyzed by (**A**) analysis of mRNA levels normalized with GAPDH; (**B**) analysis of protein levels by Western blot normalized with the β-actin standard; (**C**) observation of GFAP content by immunohistochemical analysis (scale bar 50 μm) and corresponding percentage of positive staining intensity assessed by ImageJ software (version 1.54f) (**D**). Results are expressed as mean ± SD; experiments were repeated independently in triplicate (*n* = 3) and samples with different letters indicate significant differences (*p* < 0.05) compared to own respective control cells, whereas samples sharing the same letter are not significantly different.

Based on the above results, the expression of the GFAP was analyzed in U87MG cells after treatment with 30% CGF-CM for 1, 4, and 7 days. This revealed a progressive and significant increase in both mRNA (42% at 1 day, 107% at 4 days, and 203% at 7 days) (Figure 8A) and protein levels (220% at 1 day, 413% at 4 days, and 665% at 7 days) (Figure 8B). The immunostaining with GFAP was also assessed by immunohistochemistry (Figure 8C), and the analysis of the percentage of positive staining intensity (Figure 8D) confirmed the upregulation of GFAP content observed by both gene expression and immunoblotting analyses.

**Figure 9:**
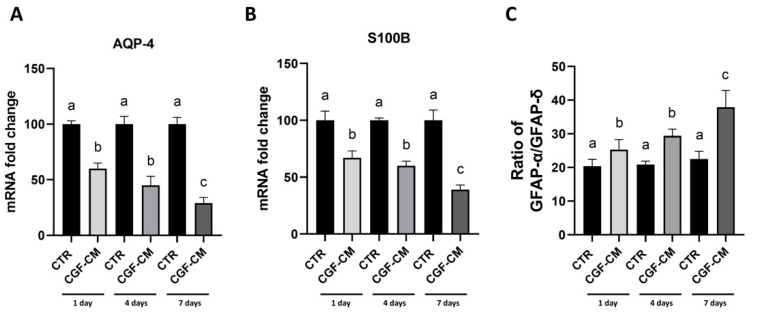
Analysis of astrocytic markers after treatment with CGF-CM. (**A**) mRNA levels of AQP-4 and S100B (**B**). (**C**) Ratio between the mRNA abundance of GFAP-α/GFAP-δ. GAPDH was used for normalization. Results are expressed as mean ± SD; experiments were repeated three times independently (*n* = 3) and samples with different letters are significantly different (*p* < 0.05) compared to own respective control cells, whereas samples sharing the same letter are not significantly different.

Based on these promising results, we considered the important markers of the astrocyte-like phenotype, such as Aquaporin 4 (AQP-4), Calcium Binding Protein B (S100B), and the ratio between the isoforms GFAP-α and GFAP-δ. The AQP-4 and S100B mRNA abundance (Figure 9A,B) showed a progressive decrease in the expression of these markers, (40% at 1 day, 55% at 4 days, 71% at 7 days for AQP-4 and 33% at 1 day, 40% at 4 days and 61% at 7 days for S100B) already evident in the first days of treatment with 30% CGF-CM and more significant at the subsequent 7-day time point. The next step was to evaluate the ratio between the levels of GFAP-α/GFAP-δ mRNA. The alpha isoform is the most abundant and predominant form of GFAP expressed in astrocytes and is often used as a marker of mature astrocytes, whereas the delta isoform plays a role in cellular plasticity [23]. From the data analysis (Figure 9C), an increase (5% at 1 day, 29% at 4 days, and 38% at 7 days) in the ratio of GFAP-α expression and a decrease in GFAP-δ expression were observed in U87MG cells treated with CGF-CM at 1, 4, and 7 days compared to their respective controls. This shift in GFAP-α/δ ratio is consistent with a transition from a highly plastic, stem-like state toward a more stable astrocytic phenotype, as described in high-grade glioma differentiation studies [23].

## 4. Discussion

CGF, a third-generation autologous platelet concentrate, has emerged as a promising tool in the field of regenerative medicine, offering significant potential for enhancing tissue repair and regeneration. Derived from the patient’s blood, CGF contains growth factors and cytokines, which are essential for cellular proliferation, differentiation, and healing [4]. The application of CGF in medical treatments leverages these biological components to accelerate wound healing and improve outcomes in bone and tissue grafting. Thanks to its features, biocompatibility, and ease of obtainment, CGF therapy is gaining attention for its efficacy and safety, paving the way for innovative treatments in orthopedics, dentistry, and beyond [24,25,26]. Previous studies on stem cells have shown that treatment with CGF promotes stem cell activation and differentiation [27,28,29,30,31,32,33].

Currently, there is a significant lack of data concerning the safe application of CGF in oncology patients. This paucity of information poses challenges for clinicians seeking to integrate CGF into treatment protocols. While the use of platelet-derived derivatives is widely documented in regenerative contexts, their interaction with tumor cells, especially in terms of phenotypic modulation, remains largely uncharacterized. This gap in knowledge is particularly relevant in GBM, a tumor characterized by high heterogeneity, plasticity, and resistance to therapies.

Panek et al. (2019) demonstrated that the local application of patches of PRF, another platelet concentrate, significantly suppresses glioma progression by inducing apoptosis in tumor cells and altering the microenvironment in a murine glioma model [22]. However, these effects are not necessarily attributable to the soluble growth factors alone, and their direct impact on tumor cell differentiation dynamics is unknown. In this context, our study sought to explore whether CGF-conditioned medium (CGF-CM) may influence GBM cell phenotype by modulating proliferation, invasiveness, and astrocytic differentiation markers.

Our first objective was to assess whether CGF-CM exerts cytotoxic or genotoxic effects on U87MG cells. The viability assay demonstrated that CGF-CM supports the metabolic activity of GBM cells in a concentration- and time-dependent manner, with a peak effect observed at 30% concentration after 4 days of treatment. This result contrasts with those of Panek et al., where PRF patches reduced the viability of glioma cells [22]; however, such differences likely reflect the distinct formats used (solid vs. soluble fractions). Importantly, further analyses confirmed that CGF-CM does not induce DNA damage or apoptosis, as evidenced by the comet assay, DAPI staining, and Caspase-3 analysis.

Beyond cell viability, we observed notable morphological changes in U87MG cells after CGF-CM treatment. Treated cells displayed a more stellate morphology, reminiscent of astrocytic features. Image-based morphometric analysis revealed a reduction in solidity and an increased perimeter-to-area ratio, consistent with enhanced morphological complexity [34]. Principal component analysis confirmed a distinct clustering of treated versus control cells, highlighting a global shift in morphometric profiles.

These findings raised the question of whether such changes reflect a transition toward a less aggressive phenotype [35]. Glioma cells are well known for their invasive potential, which is largely driven by matrix metalloproteinases (MMPs) such as MMP-2 and MMP-9 [36]. In our model, wound healing assays revealed reduced migratory capacity in CGF-CM-treated cells. This reduction in motility suggests a possible phenotypic shift toward a less invasive behavior, potentially linked to the downregulation of MMP activity or altered cytoskeletal dynamics.

Parallel evaluation of cell proliferation through growth curve analysis and cyclin D1 expression, a well-known marker promoting glioma cell proliferation [37], revealed a similar dynamic. An early increase in proliferation markers was followed by a decline at 7 days, suggesting a transient activation of cell cycle progression that resolves into a proliferative arrest. This interpretation is supported by the expression profile of cyclin D1 and phosphorylated Rb, both of which peaked at day 4 and then returned to baseline levels. Such kinetics could reflect an initial cellular adaptation to growth stimuli followed by commitment to a differentiated state.

To investigate whether this phenotypic modulation corresponded to a differentiation process, we evaluated the expression of astrocytic markers. A time-dependent increase in GFAP expression was observed at both mRNA and protein levels, indicating activation of a glial differentiation program. Immunohistochemistry further confirmed GFAP expression in CGF-CM-treated cells.

We also examined the expression of AQP-4 and S100B, which are frequently associated with glioma malignancy [38,39,40,41]. Both markers showed a progressive reduction following CGF-CM treatment. Notably, AQP-4 is implicated in glioma cell motility and invasion, and its downregulation may indicate attenuation of malignancy-related traits [39]. Similarly, S100B overexpression correlates with tumor growth and macrophage recruitment [40,41,42], and its decrease supports the hypothesis of reduced tumor aggressiveness.

Finally, analysis of the GFAP-α/GFAP-δ ratio—a marker of astrocytic maturation versus malignancy [23,43,44,45]—showed a significant shift toward the α isoform. This shift may reflect a reprogramming of GBM cells toward a more stable, differentiated astrocytic-like phenotype.

Taken together, our results suggest that CGF-CM exerts a time-dependent modulation of GBM cell phenotype, promoting a transition toward an astrocyte-like state. Rather than inducing direct cytotoxicity, CGF-CM appears to engage cellular plasticity mechanisms, suppressing proliferation and migration while enhancing differentiation markers. This model may offer a valuable platform to study non-cytotoxic modulation strategies in glioma biology.

The application of platelet-derived soluble factors to explore glioma cell differentiation opens new research avenues to investigate tumor plasticity, phenotypic switching, and the potential for differentiation-based interventions. Although speculative, these findings also support the concept that altering the tumor microenvironment through bioactive secretomes may contribute to reprogramming tumor cell behavior.

However, this study presents several important limitations. First, it relies exclusively on an in vitro model, which cannot fully reproduce the complexity of the tumor microenvironment (TME). Interactions with stromal and immune cells, vascular crosstalk, and immune modulation are absent in vitro but play a crucial role in GBM progression. Second, the study was conducted using a single GBM cell line (U87MG). Although U87MG represents a well-established and widely used experimental model, GBM is characterized by extensive inter- and intra-tumoral heterogeneity, encompassing molecular, genetic, and phenotypic variability. Therefore, results obtained in this cell line may not be fully generalizable to other GBM subtypes or patient-derived tumors. Finally, in vivo conditions may significantly alter the effects of CGF, as it contains bioactive molecules with immunosuppressive and pro-angiogenic properties, including TGF-β and VEGF. These factors may suppress antitumor immunity, recruit regulatory T cells, polarize macrophages toward a pro-tumor M2 phenotype, and promote vascularization, potentially counteracting the effects observed in vitro. Moreover, exposure to CGF-CM could impose selective pressure favoring aggressive, differentiation-resistant tumor stem cell subsets, thereby increasing the risk of tumor relapse. Another limitation of our study is that we evaluated astrocytic differentiation primarily based on morphology and marker expression without considering functional maturity, such as electrophysiological activity, neurotransmitter handling, or neuron–glia interactions. Future investigations should address these aspects in order to gain a clearer understanding of the extent to which CGF-CM induces differentiation.

Future investigations using multiple cell lines, patient-derived cultures, and in vivo models will be necessary to validate and extend the translational relevance of our findings. Therefore, while our findings highlight the phenotypic plasticity of GBM cells in response to growth factor-rich secretomes, any conclusions about therapeutic potential remain speculative, and comprehensive in vivo studies are required to validate these effects in the context of a fully functional TME.

## 5. Conclusions

In this study, we investigated the effects of a conditioned medium derived from autologous Concentrated Growth Factors (CGF-CM) on U87MG glioblastoma cells. Our findings demonstrate that CGF-CM does not induce DNA damage or apoptosis and instead modulates cell phenotype in a time-dependent manner.

Specifically, we observed an early increase in cell viability and proliferation, followed by a decrease in proliferation and migration, accompanied by morphologic and molecular markers of astrocyte-like differentiation. These effects include upregulation of GFAP and its α isoform, and downregulation of GBM -GBM-associated markers such as AQP-4 and S100B, suggesting a shift toward a less aggressive phenotype. However, it should be noted that we did not assess functional maturation, such as electrophysiological activity or glutamate secretion. Further investigation of these areas is required.

Rather than acting as a cytotoxic agent, CGF-CM appears to engage the phenotypic plasticity of GBM cells, offering a model for studying differentiation-based modulation of tumor aggressiveness. These results support the use of CGF-CM as a biological tool to explore non-genotoxic modulation of glioma cell behavior in vitro.

Future research should explore whether the observed effects can be replicated in vivo, particularly in the presence of tumor microenvironment components, and whether CGF-CM could be integrated with existing therapeutic strategies to support differentiation and/or reduce invasiveness. Overall, this study highlights the potential of platelet-derived secretomes as modulators of GBM cell plasticity and provides a foundation for further investigation into their role in tumor biology and phenotype reprogramming.

## Figures and Tables

**Table 1:** Oligonucleotides used for real-time PCR analysis.

Gene Name	Sequences (5′-3′)	Accession Number
GAPDH	F: ATGGCCTTCCGTGTCCCCAC R: ACGCCTGCTTCACCACCTTC	NM_014364.5
Cycline D1	F: TCTACACCGACAACTCCATCCG R: TCTGGCATTTTGGAGAGGAAGTG	NM_053056.3
MMP-2	F: AGCGAGTGGATGCCGCCTTTAA R: CATTCCAGGCATCTGCGATGAG	NM_001302508.1
MMP-9	F: GCCACTACTGTGCCTTTGAGTC R: CCCTCAGAGAATCGCCAGTACT	NM_004994.3
GFAP	F: CTGGAGAGGAAGATTGAGTCGC R: ACGTCAAGCTCCACATGGACCT	NM_002055.5
AQP-4	F: GCCATCATTGGAGCAGGAATCC R: ACTCAACCAGGAGACCATGACC	NM_153010
S100B	F: GGAGACGGCGAATGTGACTT R: GAACTCGTGGCAGG CAGTAGTAA	NM_006272.3
GFAP-α	F: CCCACTCTGCTTTGACTGAGC R: CCTTCTTCGGCCTTAGAGGG	NM_002055.5
GFAP-δ	F: GTGGTAAAGGTGGTGAGTCCTT R: AGAGGCTGCTGCTTGCTC	NM_001242376.3

## Data Availability

Data is contained within the article.

## Supplementary Materials

The following supporting information can be downloaded at: https://www.mdpi.com/article/10.3390/biology14101461/s1, Figure S1: Effects of CGF-CM treatment on U251 GBM cell viability. U251 GBM cells were treated with CGF-CM at 10%, 30%, or untreated cells (CTR) for 1, 4, and 7 days. Viability was determined by MTT assay. Results are expressed as mean ± SD. Experiments were repeated independently in triplicate (*n* = 3), * *p* < 0.05 compared to the same time point control cells.
