# Supplementary figures and images for: CGF-Conditioned Medium Modulates Astrocytic Differentiation and Invasiveness in U87MG Glioblastoma Cells
Source: Biology (Basel). 2025 Oct 21;14(10):1461. doi: 10.3390/biology14101461 (PMC12561328; doi:10.3390/biology14101461)

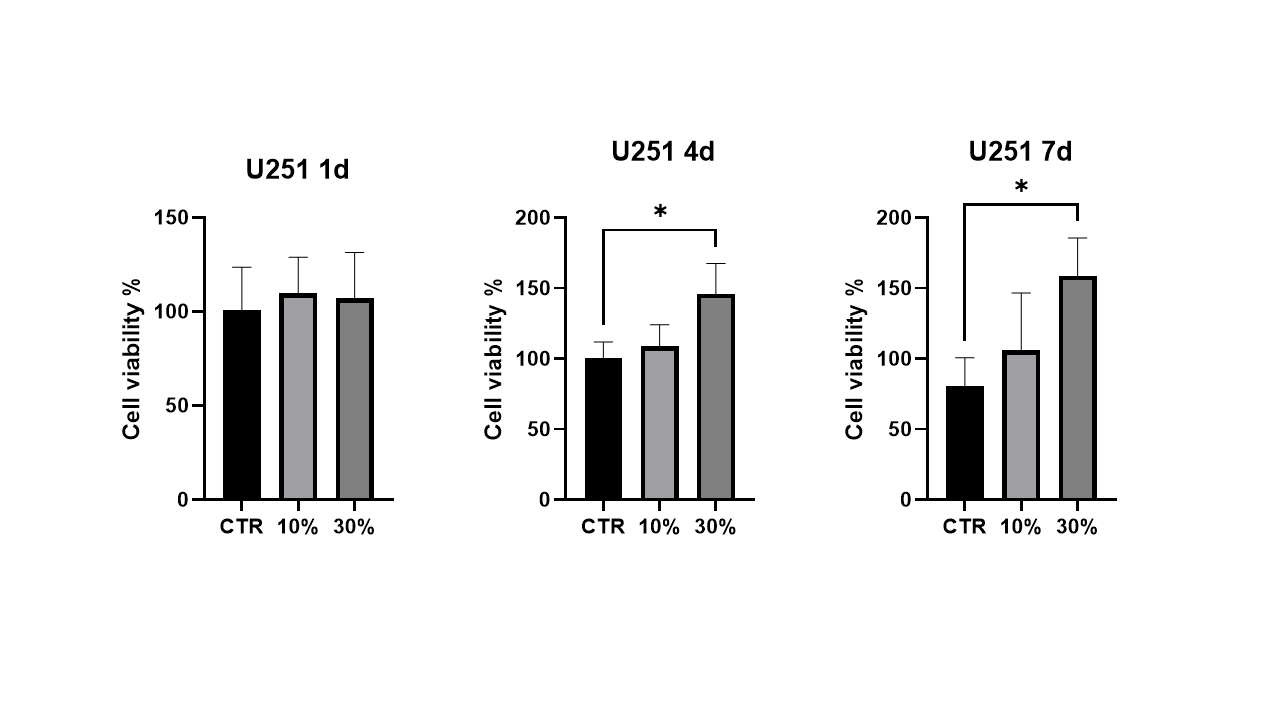

Supplement: Supplementary file 1 [file biology-14-01461-s001.zip › Figure S1 u251.png]

**Fig. 3**

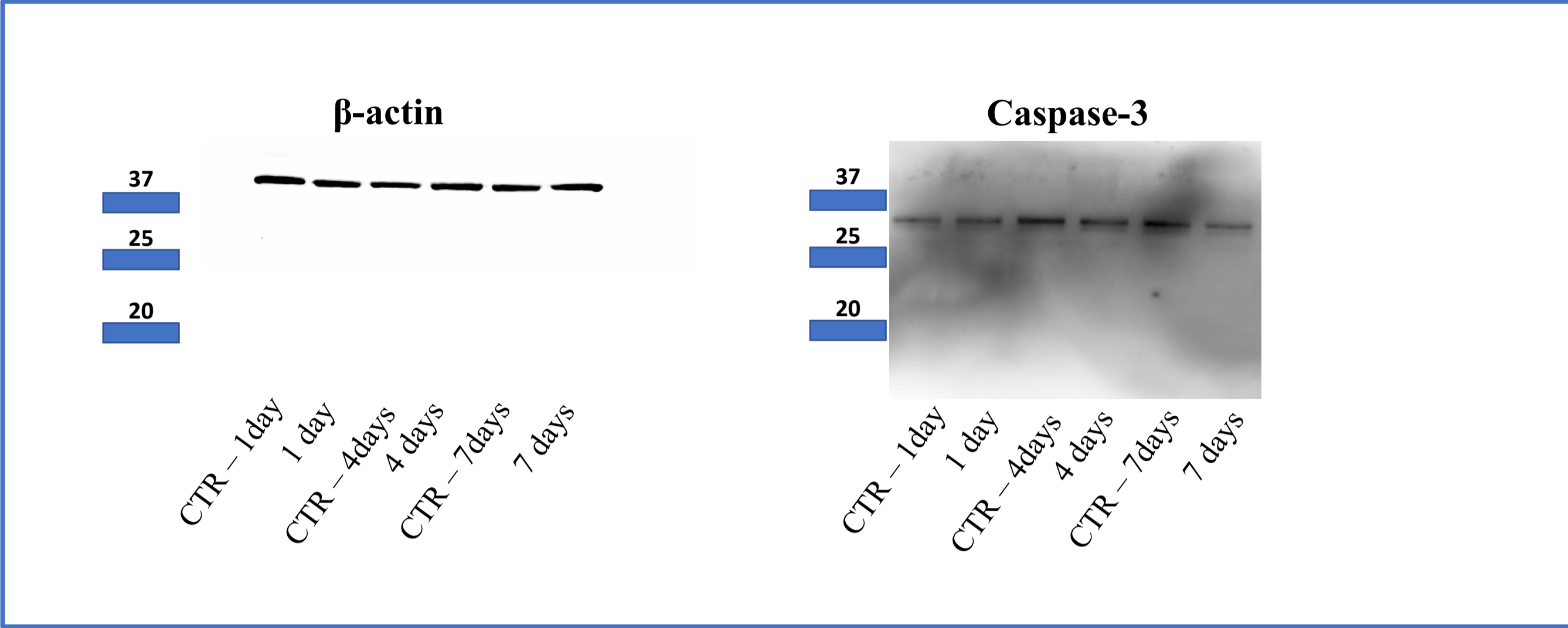

**Fig. 7**

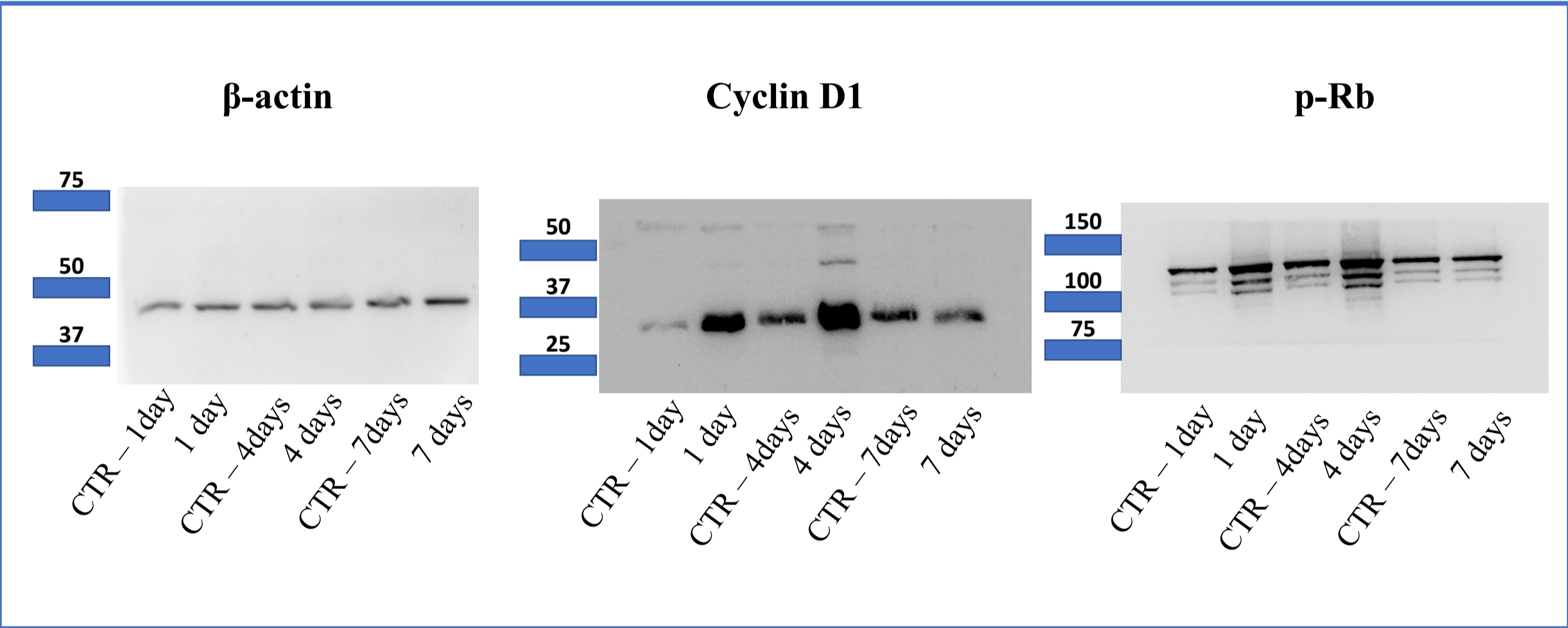

**Fig. 8**

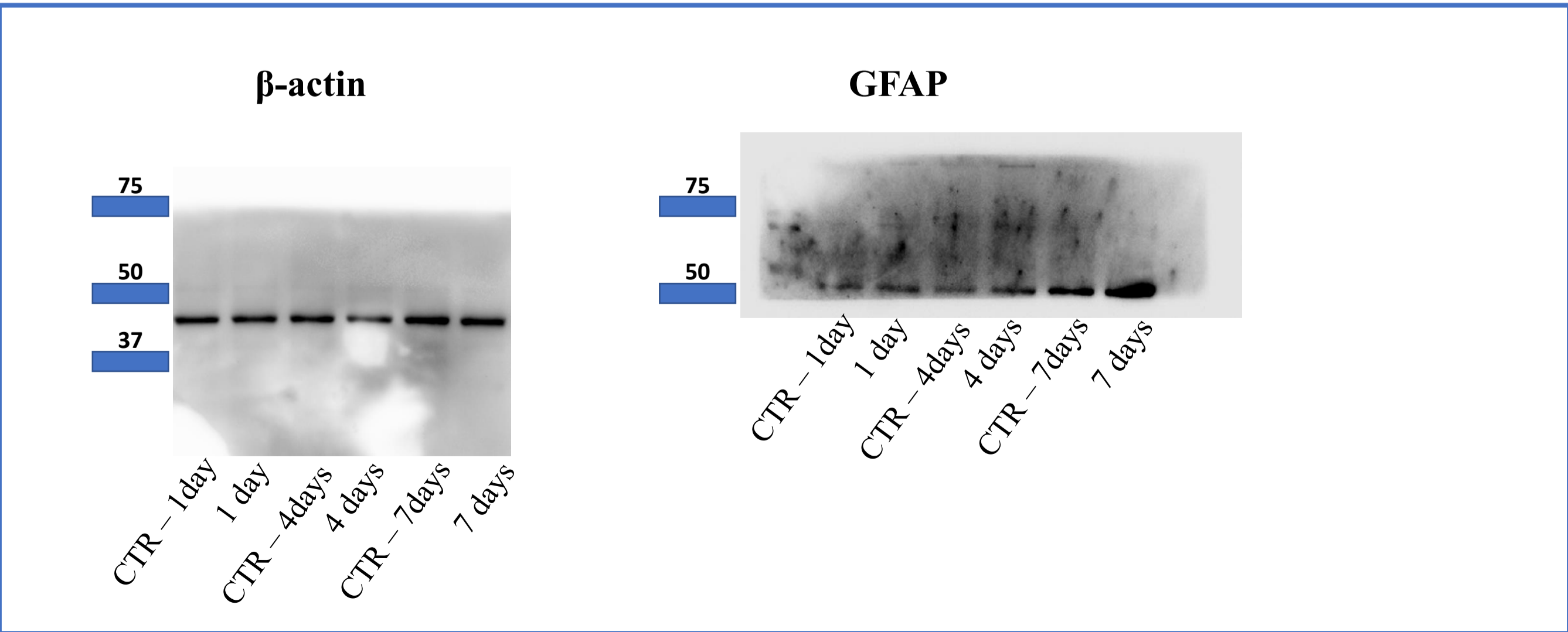

Supplement: Supplementary file 1 [file biology-14-01461-s001.zip › western completi.pdf]
